# Ruscogenin Alleviates Myocardial Ischemia-Induced Ferroptosis through the Activation of BCAT1/BCAT2

**DOI:** 10.3390/antiox11030583

**Published:** 2022-03-18

**Authors:** Fei Fu, Qiong Lai, Jingui Hu, Lu Zhang, Xiaozhou Zhu, Junping Kou, Boyang Yu, Fang Li

**Affiliations:** Jiangsu Key Laboratory of TCM Evaluation and Translational Research, Research Center for Traceability and Standardization of TCMs, School of Traditional Chinese Pharmacy, China Pharmaceutical University, Nanjing 211198, China; 3219020406@stu.cpu.edu.cn (F.F.); 1520200009@cpu.edu.cn (Q.L.); 3219020495@stu.cpu.edu.cn (J.H.); 3219020474@stu.cpu.edu.cn (L.Z.); 3320021352@stu.cpu.edu.cn (X.Z.); junpingkou@cpu.edu.cn (J.K.)

**Keywords:** myocardial ischemia, ferroptosis, ruscogenin, metabolomics, BCAT1/BCAT2, Keap1/Nrf2/HO-1 pathway

## Abstract

Ruscogenin (RUS), a natural steroidal sapogenin, exerts various biological activities. However, its effectiveness for preventing myocardial ischemia (MI) and its molecular mechanisms need further clarification. The model of MI mice and oxygen-glucose deprivation-induced cardiomyocytes injury was performed. RUS significantly alleviated MI, as evidenced by decreased infarct size, ameliorated biochemical indicators and cardiac pathological features, and markedly inhibited ferroptosis by means of the up-regulation of GPX4 and down-regulation of ACSL4 and FLC. Simultaneously, RUS notably mitigated cell injury and oxidative stress, and ameliorated ferroptosis in vitro. Subsequently, HPLC-Q-TOF/MS-based metabolomics identified BCAT1/BCAT2 as possible regulatory enzymes responsible for the cardioprotection of RUS. Importantly, RUS treatment significantly increased the expression of BCAT1 and BCAT2 in MI. Furthermore, we found that BCAT1 or BCAT2 siRNA significantly decreased cell viability, promoted ferroptosis, and increased Keap1 expression, and induced Nrf2 and HO-1 degradation in cardiomyocytes. Conversely, cardiac overexpression of BCAT1 or BCAT2 in MI mice activated the Keap1/Nrf2/HO-1 pathway. Moreover, RUS significantly activated the Keap1/Nrf2/HO-1 pathway in MI, whereas BCAT1 or BCAT2 siRNA partially weakened the protective effects of RUS, suggesting that RUS might suppress myocardial injury through BCAT1 and BCAT2. Overall, this study demonstrated that BCAT1/BCAT2 could alleviate MI-induced ferroptosis through the activation of the Keap1/Nrf2/HO-1 pathway and RUS exerted cardioprotective effects via BCAT1/BCAT2.

## 1. Introduction

Ischemic heart disease is one of the most common causes of death globally [1]. It is often caused by atherosclerotic narrowing of the coronary arteries [2]. The presence and extent of myocardial ischemia (MI) are key predictors of major adverse cardiac events, including myocardial infarction, heart failure, and death [2,3]. Although cardiovascular medical therapies have spurred a considerable reduction in mortality in recent decades, myocardial ischemia remains a major threat to human lives [3].

Various explanations exist for the pathology underlying MI, and ferroptosis has been accepted as one of the important factors [4,5]. As a recognized form of programmed cell death mode discovered in recent years, ferroptosis was characterized by intracellular iron accumulation and elevated concentration of reactive oxygen species (ROS) under high oxidative stress [6,7]. In particular, glutathione peroxidase 4 (GPX4), which protects cells from ferroptosis, was down-regulated in the stages of MI [5]. Additionally, previous studies identified acyl-CoA synthetase long-chain family member 4 (ACSL4) and ferritin as key targets in the regulation of ferroptosis [8]. GPX4 depletion and accumulated ACSL4 in cells will result in the accumulation of lipid peroxides. Additionally, ferritin modulates sensitivity to ferroptosis through its impact on iron metabolism [5,8]. Furthermore, accumulative evidence demonstrated that the activation of the nuclear factor E2-related factor 2 (Nrf2) signaling was involved in promoting resistance to ferroptosis [9]. Kelch-like ECH-associated protein 1 (Keap1) binds to Nrf2 in the cytoplasm and is responsible for the ubiquitination of Nrf2. When cells are suffering oxidative stress, Nrf2-dependent cellular defense mechanisms are opened, and Nrf2 separates from Keap1. This allows Nrf2 to escape ubiquitination, accumulate within the cell, and translocate to the nucleus [10], where Nrf2 initiates the transcription of downstream antioxidant genes, such as quinone oxidoreductase 1, glutamate-cysteine ligase, heme oxygenase-1 (HO-1), etc. In particular, HO-1 has been used as a hallmark Nrf2 target gene and a major antioxidant enzyme for protecting cells from further oxidative insult [11]. The Keap1/Nrf2/HO-1 pathway is positioned to be a central inhibitory pathway of ferroptosis in anti-cancer therapies, as well as the prevention of neurodegenerative and cardiovascular diseases [12,13,14].

Ruscogenin (RUS), an important active ingredient found in the root of *Ophiopogon japonicus*, was applied in the treatment of acute and chronic inflammation [15]. Previous studies have documented that RUS exerted beneficial effects on alleviating pulmonary arterial hypertension and acute lung injury, as well as on the prevention of blood–brain barrier dysfunction [15,16,17]. Nevertheless, the cardioprotective effect of RUS remains to be further clarified. Metabolomics, the study of small-molecule metabolites and metabolic pathways in biological systems, has been developed as a powerful tool to diagnose diseases, elucidate the complex pathophysiologic processes, and identify new drug targets [18,19]. The functional metabolomics revealed that the downregulation of *N*-acetylneuraminic acid by silencing neuraminidase-1 represents an effective therapeutic strategy in the treatment of coronary artery disease [20], whereas the cardioprotective mechanism of RUS from the perspective of metabolomics has not been reported.

This study was performed to elucidate the cardioprotective effect of RUS in MI mice and oxygen-glucose deprivation (OGD)-injured H9c2 cardiomyocytes. Metabolomics was further applied to clarify the metabolic pathways and key enzymes regulated by RUS, and the potential mechanism was further verified. Our results will provide a promising strategy for the prevention and treatment of MI and lay the foundation for the further application of RUS.

## 2. Materials and Methods

### 2.1. Chemicals and Reagents

Methyl thiazolyl tetrazolium (MTT) was purchased from Amresco (Washington, DC, USA). RUS was purchased from J&K Scientific Ltd. (Beijing, China; purity: HPLC ≥ 98%). *N*-acetyl-L-cysteine (NAC) was obtained from Sigma-Aldrich (St. Louis, MO, USA). Metoprolol (Met) tartrate tablets were purchased from AstraZeneca (Taizhou, China). 2,3,5-triphenyl tetrazolium chloride (TTC) was purchased from Macklin Biochemical Co., Ltd. (Shanghai, China). Serum creatine kinase (CK), serum lactate dehydrogenase (LDH), and glutathione (GSH) assay kits were obtained from Nanjing Jiancheng Bioengineering Institute (Nanjing, China). Cell lactate dehydrogenase, total superoxide dismutase (SOD) with the WST-8 method, malondialdehyde (MDA), and ROS assay kits were attained commercially from Beyotime Biotechnology Institute (Shanghai, China). Iron colorimetric assay kits were purchased from Applygen Technologies (Beijing, China). Antibodies against GAPDH were purchased from Bioworld Technology (St. Louis Park, MN, USA). Antibodies against β-actin and GPX4 were purchased from ZenBio (Chengdu, China). Antibodies against ACSL4 were purchased from ABclonal Technology (Wuhan, China). Antibodies against branched-chain amino acid aminotransferase 1/2 (BCAT1/BCAT2), Nrf2, Keap1, HO-1 and ferritin light chain (FTL) were obtained commercially from Proteintech (Wuhan, China).

### 2.2. Animal Experiment

ICR male mice (8 weeks old, 22–25 g) were purchased from the Model Animal Research Centre of Yangzhou University (Yangzhou, China, certificate No. 202107969) and housed in standard laboratory conditions. All animal procedures followed the National Institutes of Health Guide for the Care and Use of Laboratory Animals, and the experimental protocols were approved by the Animal Ethics Committee of China Pharmaceutical University (Nanjing, China, approval No. SYXK 2021-0011). The MI model was induced by left anterior descending coronary artery ligation (CAL). Sham-operated mice underwent the same procedures, except for the ligation. The mice were randomly separated into six groups (*n* = 12): (i) the sham group; (ii) the MI group; (iii) the low dose of RUS group (0.375 mg/kg, i.p.); (iv) the middle dose of RUS group (0.75 mg/kg, i.p.); (v) the high dose of RUS group (1.5 mg/kg, i.p.); (vi) the Met group (5.14 mg/kg, i.g.). RUS was dissolved in absolute ethyl alcohol at 30 mg/mL and stored at −20 °C. For intraperitoneal injection, the stock solution was diluted in normal saline at different concentrations. All drugs were administered in 20 min after CAL. Twenty-four hours after the surgery, all mice were euthanized. 

### 2.3. TTC Staining

The hearts were removed and immediately perfused with phosphate buffer saline (PBS), frozen at −70 °C and then transversely cut into five slices. The slices were stained with 1% TTC at 37 °C for 15 min. The white area of the heart negatively stained by TTC represented infarcted tissue. 

### 2.4. Blood Sample Analysis

Twenty-four hours after CAL, blood samples were collected by puncturing the retro-orbital venous plexus of the mice and centrifuged at 3500 rpm for 10 min to obtain serum samples. To assess the myocardial damage, the levels of CK and LDH in serum were measured using commercial kits as directed by the manufacturer. 

### 2.5. Histopathologic Examination

The heart tissues were perfusion-fixed with 4% paraformaldehyde solution, embedded in paraffin wax, and sliced into 4–5 µm histological sections. Then, the heart sections were stained with hematoxylin-eosin (HE), which was applied to investigate the morphological damages. The histopathological score analysis was conducted by two pathologists blinded to the animal groups referring to previous research [21]. 

### 2.6. Cell Culture and OGD Injury

Rat H9c2 cardiomyocyte cell line purchased from Shanghai Institute of Cell Biology, Chinese Academy of Science (Shanghai, China) was cultured in Dulbecco’s modified Eagle medium (DMEM) supplemented with 10% fetal bovine serum (FBS, Gibco, Rockville, MD, USA) and 1% penicillin and streptomycin (Beyotime, Shanghai, China) at 37 °C with 5% CO_2_. To mimic the OGD injury, H9c2 cardiomyocytes were incubated with FBS-free and glucose-free DMEM and exposed to a hypoxic environment of 94% N_2_, 5% CO_2_ and 1% O_2_ at 37 °C. Meanwhile, different concentrations of RUS (0.1, 1, 10 μM) or 500 μM NAC (positive control drug) were dissolved in the culture medium of OGD-treated cells.

### 2.7. Measurement of Cell Viability and LDH

Cell viability was determined through the MTT assay. After different treatments in 96-well plates, cells were incubated with 0.5 mg/mL MTT for 3 h at 37 °C. After removing the medium, 150 μL of dimethylsulfoxide was added to dissolve the formazan crystals. The optical density (OD) for each well was measured at 570 nm with a reference wavelength of 650 nm. The release of LDH was also tested to further measure the level of cell injury. After the incubation, the culture supernatants were collected and used to detect the OD at 490 nm according to the manufacturer’s instructions. 

### 2.8. Determination of GSH, SOD, MDA and Iron Levels

H9c2 cardiomyocytes were washed with ice-cold PBS, and then the cells were crushed. The contents of GSH, SOD, MDA and total Fe in H9c2 cardiomyocytes were measured using commercial assay kits as directed by the manufacturer. GSH, SOD, MDA and total Fe in the cells were detected at the absorption wavelengths of 405 nm and 450 nm with a reference wavelength of 600 nm, 532 nm and 550 nm, respectively.

### 2.9. Measurement of ROS

Intracellular ROS was detected using fluorescent 2′,7′-dichlorofluorescin diacetate (DCFH-DA, Beyotime, Shanghai, China) as a probe. Cells were seeded into small confocal plates and treated with OGD, RUS and NAC. The culture medium was removed, then the cells were washed with PBS and incubated with 10 µM DCFH-DA diluted in FBS-free medium for 20 min at 37 °C. Subsequently, the cells were washed with an FBS-free medium. ROS levels were measured by a confocal laser scanning microscopy (CLSM, LSM700, Zeiss, Germany).

### 2.10. Untargeted Metabolomics Analysis

Samples of urine and blood were collected after ligation for 24 h. Sample preparation procedures refer to previous protocols [22]. An Agilent Technologies 6530 UPLC system coupled with a Q-TOF MS instrument (Santa Clara, CA, USA) was employed for metabolite separation. MassHunter Workstation software and R package were used for data acquisition and pretreatment. Then, the processed data were inputted into SIMCA-P 14.1 software (Umetrics, Sweden) for principal component analysis (PCA) and orthogonal partial least squares discriminant analysis (OPLS-DA). The data were further imported into the MetaboAnalyst 5.0 platform (https://www.metaboanalyst.ca/ (accessed on 30 April 2021)) for univariate analysis. To identify potential metabolites, the messages of metabolites were matched in Metlin (http://metlin.scripps.edu/ (accessed on 25 April 2021)) and Human Metabolome Database (http://www.hmdb.ca/ (accessed on 25 April 2021)). Then, the online platforms including MetaboAnalyst 5.0 and STRING (https://string-db.org/ (accessed on 30 April 2021)) were implemented for the enrichment and pathway analysis of influential metabolites and related regulated enzymes.

Measurement of quality control (QC) was performed for QC samples. The results of unsupervised PCA analysis show that the analytical procedure had good reproducibility and stability (Figure S1). In addition, the relative standard deviation (RSD) of retention times and peak areas was below 1% and 5%, respectively (Table S1). These data indicated that the reproducibility of the method and stability of the equipment were good. 

### 2.11. Immunofluorescence Staining

The heart tissues were immersed in 4% paraformaldehyde solution, dehydrated, and sliced into 4–5 μm-thick pieces. The tissues were permeabilized with 0.1% Triton X-100, subsequently blocked with 5% bovine serum albumin (BSA) for 30 min, then incubated with a rabbit GPX4 antibody at 1:100 dilution overnight at 4 °C. For cultured cells, they were washed with ice-cold PBS, permeabilized, fixed, blocked, and incubated with primary antibodies against BCAT1 (1:50 dilution), BCAT2 (1:50 dilution), Nrf2 (1:50 dilution) and HO-1 (1:20 dilution) overnight at 4 °C, followed by incubation with Dylight 488 donkey anti-rabbit secondary antibody (Bioworld Technology, Louis Park, MN, USA) and DAPI (Beyotime Biotechnology, Shanghai, China). MitoTracker^®^ Deep Red (Molecular Probes, Invitrogen, Carlsbad, CA, USA) and Alexa Fluor 568 Phalloidin (Invitrogen, Carlsbad, CA, USA) were used for mitochondria and F-actin staining, respectively. Fluorescent images were observed using a confocal laser scanning microscopy.

### 2.12. Immunohistochemistry 

The expression of BCAT1, BCAT2, Keap1, Nrf2 and HO-1 was analyzed by means of immunohistochemistry. The 5 μm tissue sections of paraffin-embedded hearts were deparaffinized, hydrated, incubated with 3% peroxide-methanol and then blocked for 1 h. The primary antibodies were dropwise added to the heart section overnight at 4 °C, followed by the incubation with the HRP-conjugated secondary antibodies (1:200, Biogot Technology, Nanjing, China) for 1 h at room temperature. After incubation with 3,3′-diaminobenzidine and counterstaining with hematoxylin, a NanoZoomer 2.0 RS (Hamamatsu, Japan) was conducted to scan the heart section.

### 2.13. siRNA Transfection

H9c2 cardiomyocytes were transfected with appropriate siRNA against BCAT1 (75 nmol/L) or BCAT2 (75 nmol/L) or 75 nmol/L negative control (NC), synthesized by Biomics Biotechnologies Co., Ltd. (Nantong, China). Transfection in cells was performed using Hifect transfection reagent (Nanjing ICre Bioscience Technology, Nanjing, China). The transfection efficiency was estimated by Western blot analysis after transfection for 48 h. The siRNA sequences are listed in Table S2.

### 2.14. Infection of Adeno-Associated Virus

In vivo infection, adeno-associated virus (AAV) overexpressing BCAT1 or BCAT2, and control virus particles (×10 pfu/mL, Genomeditech company, Shanghai, China) were directly injected into the left ventricular free wall (2 sites, 10 μL/site) in mice. Two weeks later, the sham and CAL surgeries were performed. The efficiency of gene transduction in cryosectioned heart slices was assessed by EGFP fluorescence. 

### 2.15. Western Blot Analysis

Heart tissues from infarct areas were collected, and then crushed by a tissue homogenizer. Cultured cells were washed with ice-cold PBS, and collected by a cell scraper. Both the tissues and cells were lysed by ice-cold RIPA buffer supplemented with 1% PMSF for 30 min, then centrifuged at 12,000 rpm for 10 min at 4 °C. The protein concentrations were detected by means of the BCA method. Equal amounts of proteins were loaded onto SDS-PAGE for electrophoresis and subsequently transferred onto the activated PVDF membranes. The membranes were blocked with 5% BSA and probed with primary antibodies against GPX4, ACSL4, FTL, BCAT1, BCAT2, Keap1, Nrf2, HO-1, GAPDH, and β-actin (dilution 1:1000, 1:500, 1:1000, 1:1000, 1:500, 1:2000, 1:1000, 1:1000, 1:8000, 1:10,000) overnight at 4 °C. Membranes were then probed with peroxidase-conjugated secondary antibody at 1:10,000 dilution (Bioworld Technology, Louis Park, MN, USA). The protein signal was detected using the ECL plus system (Amersham, Arlington Heights, IL, USA) and the band densities were determined by Bio-Rad Laboratories. 

### 2.16. Statistical Analysis

Statistical analysis was carried out using GraphPad Prism 8 software. All data in the present study are presented as the mean ± SD. Differences between two groups were analyzed by Student’s two-tailed t-test, and one-way analysis of variance followed by Dunnett’s test was used for comparisons among multiple groups. *p* < 0.05 was considered statistically significant. 

## 3. Results

### 3.1. RUS Effectively Alleviated Myocardial Injury and Inhibited Ferroptosis in MI Mice

**Figure 1:**
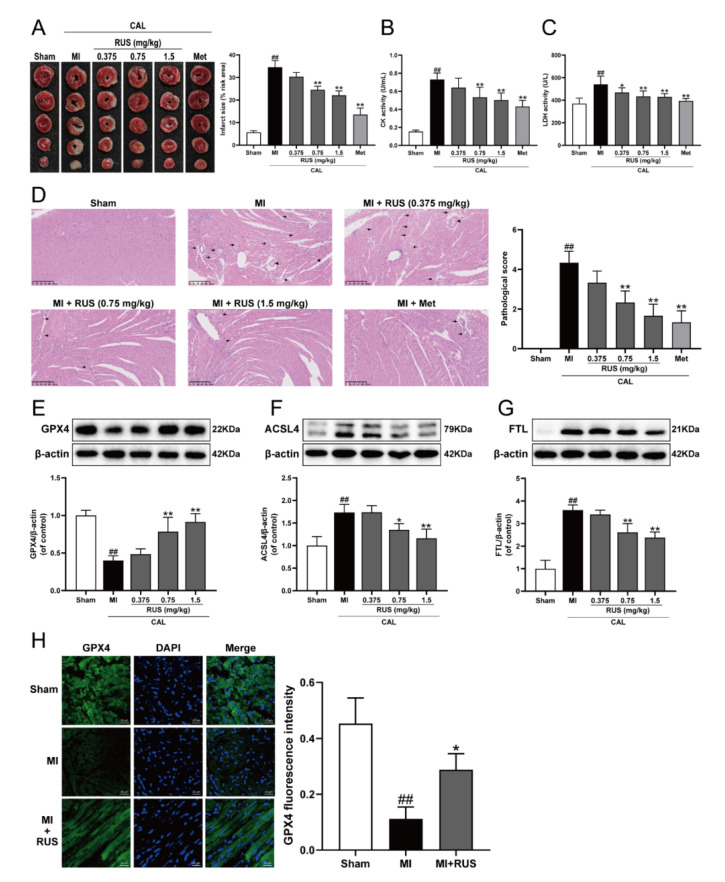
RUS effectively alleviated myocardial injury and inhibited ferroptosis in MI mice. (**A**) TTC staining images of the heart infarct size (*n* = 3). The serum activity of (**B**) CK and (**C**) LDH (*n* = 6). (**D**) HE staining images of the heart tissues (scale bar, 250 μm, *n* = 3). Histopathological changes are indicated by arrows (inflammatory cellular infiltration) and arrowheads (hyperemic blood vessels). Western blotting analysis of (**E**) GPX4, (**F**) ACSL4, and (**G**) FTL in the heart tissues (*n* = 3–4). (**H**) Immunofluorescence images of GPX4 in the heart tissues of sham, MI and MI treated with 0.75 mg/kg RUS groups (scale bar, 20 µm, *n* = 3). Results were expressed as mean ± SD. ^##^ *p* <  0.01 vs. the sham group, * *p*  <  0.05, ** *p * <  0.01 vs. the MI group.

As shown in Figure 1A–C, 24 h of CAL resulted in myocardial infarction, as evidenced by the significantly augmented infarct size, elevated serum contents of CK and LDH, whereas Met treatment and RUS with the dose of 0.75 mg/kg and 1.5 mg/kg significantly attenuated the CAL-induced myocardial impairment. In addition, in the results of HE staining, heart tissue sections of the RUS groups and Met group showed decreased myocardial structural disarray and morphological damage compared with the MI group (Figure 1D). As shown in Figure 1E–G, RUS significantly enhanced the level of GPX4, and inhibited the expression of ACSL4 and FTL. Immunofluorescence analysis also indicated that RUS at the dose of 0.75 mg/kg up-regulated GPX4 expression (Figure 1H). All these results reveal the effects of cardioprotection and anti-ferroptosis of RUS in MI mice.

### 3.2. RUS Significantly Protected OGD-Injured H9c2 Cardiomyocytes and Inhibited Oxidative Stress and Ferroptosis

**Figure 2:**
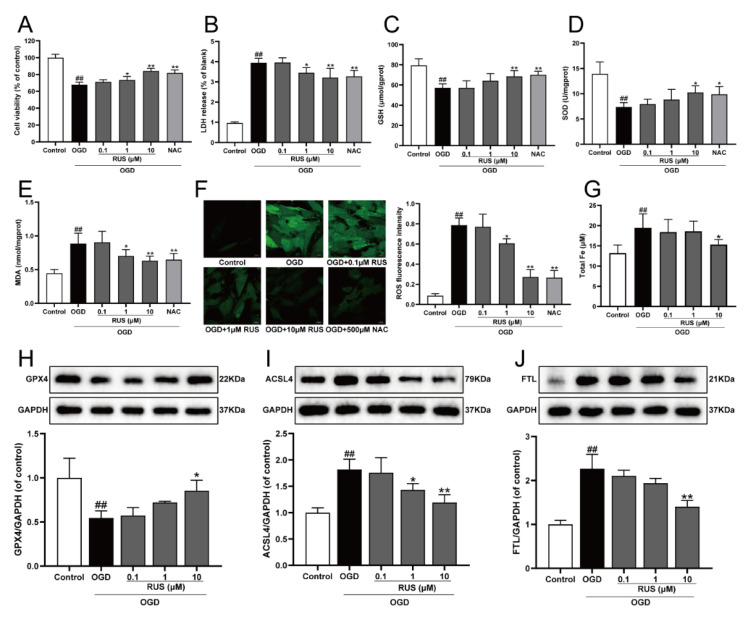
RUS significantly protected OGD-injured H9c2 cardiomyocytes and inhibited oxidative stress and ferroptosis. (**A**) The cell viability of H9c2 cardiomyocytes (*n* = 6). (**B**) The LDH release of H9c2 cardiomyocytes (*n* = 6). The contents of (**C**) GSH, (**D**) SOD and (**E**) MDA in H9c2 cardiomyocytes (*n* = 6). (**F**) Immunofluorescence analysis of ROS production in H9c2 cardiomyocytes (scale bar, 20 µm, *n* = 3). (**G**) The content of total iron in H9c2 cardiomyocytes (*n* = 6). Representative Western blotting analysis of (**H**) GPX4, (**I**) ACSL4, and (**J**) FTL in H9c2 cardiomyocytes (*n* = 3–4). Results were expressed as mean ± SD. ^##^ *p* <  0.01 vs. the control group, * *p*  <  0.05, ** *p * <  0.01 vs. the OGD group.

The level of ferroptosis in H9c2 cardiomyocytes gradually enhanced with the prolongation of OGD-injured time. Additionally, the expression of both GPX4 and ACSL4 was both significantly changed at 6 h of OGD treatment, and this expression was selected for further study (Figure S2). As exhibited in Figure 2A,B, the viability of H9c2 cardiomyocytes subjected to 1–10 μM RUS was dramatically increased, and RUS treatment markedly inhibited the release of LDH. Moreover, OGD injury resulted in devitalized GSH and SOD, and accumulated MDA and ROS, which were adverse phenomena of oxidative stress and indirectly reflected cell damage. However, RUS significantly ameliorated OGD-induced oxidative injury in cardiomyocytes (Figure 2C–F). As shown in Figure 2G, 10 μM RUS diminished the intracellular iron accumulation. In addition, 10 μM RUS up-regulated the expression of GPX4, and significantly down-regulated ACSL4 and FTL in OGD-injured H9c2 cardiomyocytes (Figure 2H–J). Collectively, these results suggest that RUS effectively inhibited OGD-induced oxidative stress and ferroptosis in H9c2 cardiomyocytes.

### 3.3. Multivariate Analysis and Metabolic Pathway Analysis

**Figure 3:**
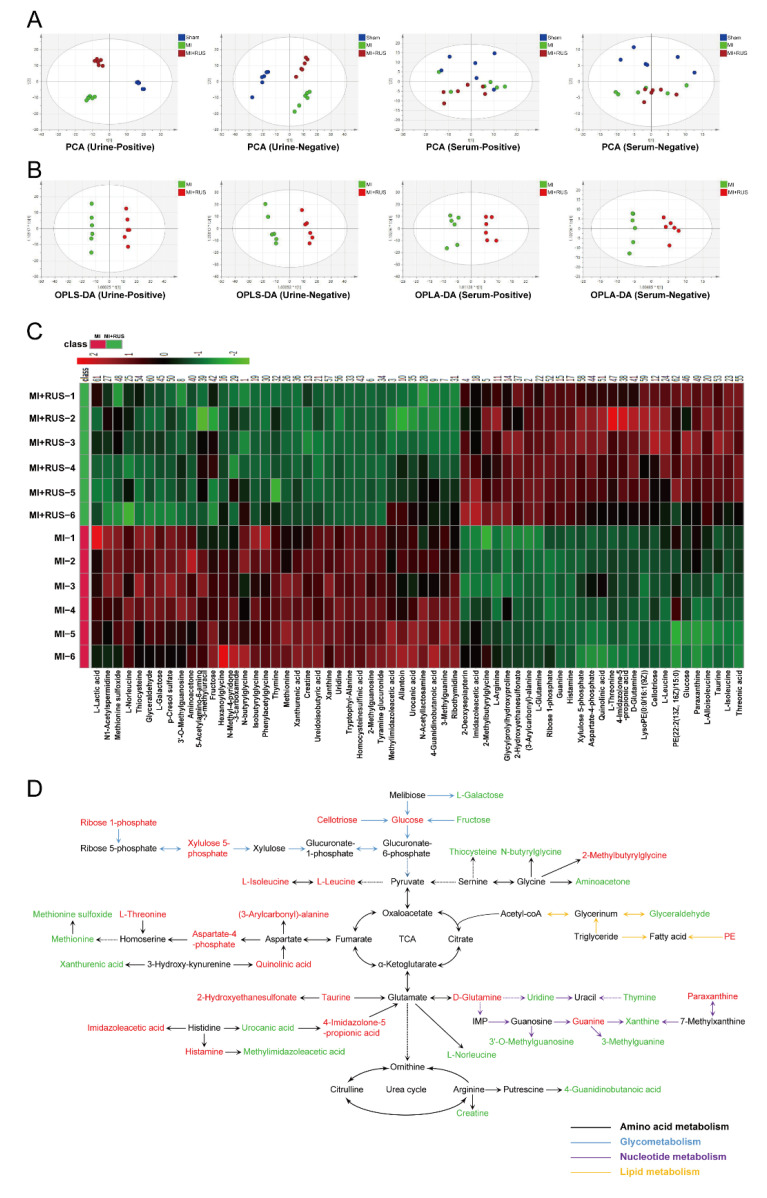
Multivariate analysis and metabolic network of the significantly changed endogenous metabolites. (**A**) PCA score plots of the sham, MI, and MI treated with 0.75 mg/kg RUS groups. (**B**) OPLS-DA score plots of the MI and MI treated with 0.75 mg/kg RUS groups. (**C**) Heat map of 62 differential endogenous metabolites in urine and serum (*n* = 6). (**D**) The metabolic network map of the differential metabolites and their changed metabolic pathways. Red metabolites in the network are increased in the RUS group compared with the MI group and the down-regulated metabolites are represented by green.

As shown in Figure 3A, the results of PCA analysis demonstrate a clear separation of metabolic profiling among sham, MI, and RUS groups. The supervised OPLS-DA was adopted to evaluate the alterations of metabolome between RUS and MI groups (Figure 3B). The high model quality parameters of R^2^Y and Q^2^ demonstrated the good fitness and prediction power of OPLS-DA (Table S3). As shown in Tables S4 and S5, 58 differentially expressed metabolites in urine and 4 metabolites in serum were selected and identified as potential biomarkers. Furthermore, a heat map was constructed to give a more visualized view (Figure 3C). Additionally, according to the fold enrichment of pathways, multiple metabolic pathways were impaired after RUS treatment (Figure S3A–C). Additionally, the network map of protein interaction and the result of GO enrichment analysis are shown in Figure S3D,E. Meanwhile, the Kyoto Encyclopedia of Genes and Genomes (KEGG) enrichment analysis suggested that the related regulatory enzymes of RUS were principally involved in histidine metabolism and valine, leucine and isoleucine biosynthesis (Table 1). According to the above results, a metabolic network map of the differential metabolites involving amino acid metabolism, glycometabolism, nucleotide metabolism, lipid metabolism was constructed (Figure 3D). RUS may ameliorate MI by affecting the above metabolic pathways.

### 3.4. RUS Significantly Promoted the Expression of BCAT1/BCAT2 in MI

**Figure 4:**
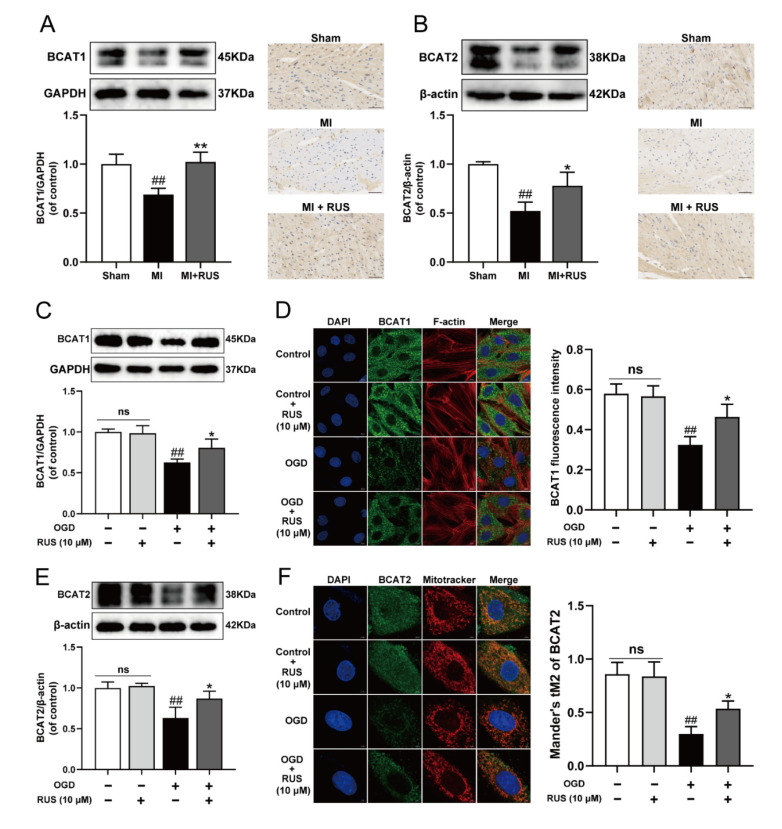
RUS significantly promoted the expression of BCAT1/BCAT2 in MI. Western blotting and immunohistochemistry analysis of (**A**) BCAT1 and (**B**) BCAT2 in the heart tissues of the sham, MI and MI treated with 0.75 mg/kg RUS groups (scale bar, 50 µm, *n* = 3). (**C**) Western blotting analysis of BCAT1 in H9c2 cardiomyocytes (*n* = 3). (**D**) Immunofluorescence images of BCAT1 in H9c2 cardiomyocytes (scale bar, 10 µm, *n* = 3). (**E**) Western blotting analysis of BCAT2 in H9c2 cardiomyocytes (*n* = 3). (**F**) Immunofluorescence images of BCAT2 in H9c2 cardiomyocytes (scale bar, 5 µm, *n* = 3). Results were expressed as mean ± SD. ^##^ *p*  <  0.01 vs. the sham or control group, * *p*  <  0.05, ** *p * <  0.01 vs. the MI or OGD group.

The BCAT1 and BCAT2 proteins highly ranking in KEGG results might play an important role in the regulation of metabolic pathway by RUS to improve myocardial ischemia. We found that the expression of BCAT1 and BCAT2 gradually declined during OGD treatment (Figure S4). Then, the regulatory effect of RUS on BCAT1 and BCAT2 was further investigated. As illustrated in Figure 4A,B, RUS at the dose of 0.75 mg/kg markedly increased the levels of BCAT1 and BCAT2 in MI mice. The relevant statistical results of immunohistochemistry analysis were shown in Figure S5. Meanwhile, 10 μM RUS treatment increased the expression of BCAT1 and BCAT2, and enhanced the colocalization of the mitochondria with BCAT2 in OGD-injured H9c2 cardiomyocytes (Figure 4C–F). However, RUS did not affect the expression of BCAT1 and BCAT2 in H9c2 cardiomyocytes without OGD injury.

### 3.5. Knockdown of BCAT1 or BCAT2 Aggravated Ferroptosis and Restrained Keap1/Nrf2/HO-1 Signaling Pathway in H9c2 Cardiomyocytes

**Figure 5:**
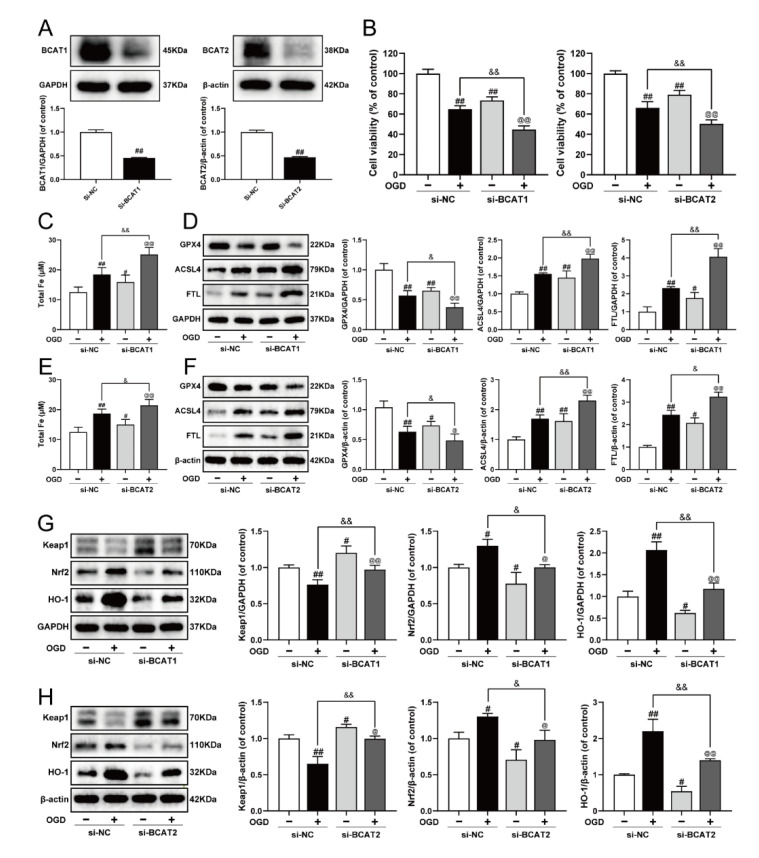
Knockdown of BCAT1 or BCAT2 aggravated ferroptosis and restrained the Keap1/Nrf2/HO-1 signaling pathway in H9c2 cardiomyocytes. (**A**) Western blotting analysis of BCAT1 and BCAT2 in H9c2 cardiomyocytes transfected with BCAT1 siRNA or BCAT2 siRNA (*n* = 3–4). (**B**) The cell viability of H9c2 cardiomyocytes transfected with BCAT1 siRNA or BCAT2 siRNA (*n* = 6). (**C**) The content of total iron of H9c2 cardiomyocytes transfected with BCAT1 siRNA (*n* = 6). (**D**) Western blotting analysis of GPX4, ACSL4, and FTL in H9c2 cardiomyocytes transfected with BCAT1 siRNA (*n* = 3). (**E**) The content of total iron of H9c2 cardiomyocytes transfected with BCAT2 siRNA (*n* = 6). (**F**) Western blotting analysis of GPX4, ACSL4, and FTL in H9c2 cardiomyocytes transfected with BCAT2 siRNA (*n* = 3). Western blotting analysis of Keap1, Nrf2 and HO-1 in H9c2 cardiomyocytes transfected with (**G**) BCAT1 siRNA or (**H**) BCAT2 siRNA (*n* = 3). Results were expressed as mean ± SD. ^#^ *p*  <  0.05, ^##^ *p*  <  0.01 vs. the control group, ^&^ *p*  <  0.05, ^&&^ *p * <  0.01 vs. the OGD group, ^@^ *p*  <  0.05, ^@@^ *p * <  0.01 vs. the group treated with BCAT1 siRNA or BCAT2 siRNA alone.

Previously, we elucidated that BCAT1/BCAT2 might be a promising target for the clinical management of MI, and was closely associated with myocardial ferroptosis [23]. In the present study, siRNA was used to further clarify the molecular mechanism. As exhibited in Figure 5A–F, BCAT1 or BCAT2 siRNA decreased cell viability, and evoked ferroptosis by augmenting iron level, inhibiting GPX4, as well as increasing the expression of ACSL4 and FTL in OGD-injured cardiomyocytes. Meanwhile, BCAT1 or BCAT2 siRNA also obviously inhibited cell viability and promoted ferroptosis in cardiomyocytes without OGD injury. Keap1/Nrf2/HO-1 is an important ferroptosis-related signaling pathway implicated in defense mechanisms against oxidative injury, and its activation is a common feature of inhibiting ferroptosis [9,10]. Compared with the OGD group, BCAT1 or BCAT2 siRNA inhibited the Keap1/Nrf2/HO-1 signaling pathway, as evidenced by significantly decreased levels of Nrf2 and HO-1 and the upregulation of Keap1. Simultaneously, BCAT1 or BCAT2 siRNA also inhibited the Keap1/Nrf2/HO-1 signaling pathway in cardiomyocytes without OGD injury (Figure 5G,H). These results indicate that BCAT1 or BCAT2 siRNA significantly promoted ferroptosis and restrained the Keap1/Nrf2/HO-1 pathway in H9c2 cardiomyocytes. 

### 3.6. Cardiac Overexpression of BCAT1 or BCAT2 Activated the Keap1/Nrf2/HO-1 Signaling Pathway in MI Mice

**Figure 6:**
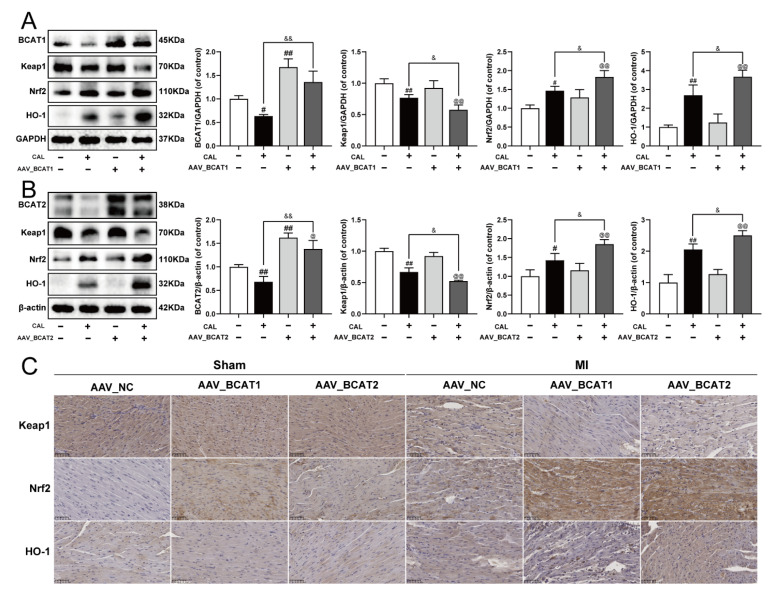
Cardiac overexpression of BCAT1 or BCAT2 activated the Keap1/Nrf2/HO-1 signaling pathway in MI mice. (**A**) Western blotting analysis of BCAT1, Keap1, Nrf2 and HO-1 in mice with cardiac overexpression of BCAT1 (*n* = 3–4). (**B**) Western blotting analysis of BCAT2, Keap1, Nrf2 and HO-1 in mice with cardiac overexpression of BCAT2 (*n* = 3–4). (**C**) Immunohistochemistry images of Keap1, Nrf2 and HO-1 in mice with cardiac overexpression of BCAT1 or BCAT2 (scale bar, 50 µm, *n* = 3). Results were expressed as mean ± SD. ^#^ *p*  <  0.05, ^##^ *p*  <  0.01 vs. the sham group, ^&^ *p*  <  0.05, ^&&^ *p * <  0.01 vs. the MI group, ^@^ *p * <  0.05, ^@@^ *p * <  0.01 vs. the group treated with AAV-BCAT1 or AAV-BCAT2.

To further elucidate the possible molecular mechanism of BCAT1/BCAT2 in attenuating myocardial ferroptosis, the cardiac overexpression of BCAT1 or BCAT2 was constructed by intramyocardial injection of AAV-BCAT1 or AAV-BCAT2 in mice. As shown in Figure 6A–C, the cardiac overexpression of BCAT1 or BCAT2 reduced the expression of Keap1, while the levels of Nrf2 and HO-1 were augmented. However, cardiac overexpression of BCAT1 or BCAT2 had no significant effect on the Keap1/Nrf2/HO-1 signaling pathway in sham-operated mice. The relevant statistical results of immunohistochemistry analysis were also shown in Figure S6. All these results suggest that BCAT1 and BCAT2 were positive regulators of the Keap1/Nrf2/HO-1 signaling pathway in MI mice.

### 3.7. RUS Significantly Activated the Keap1/Nrf2/HO-1 Signaling Pathway in MI

**Figure 7:**
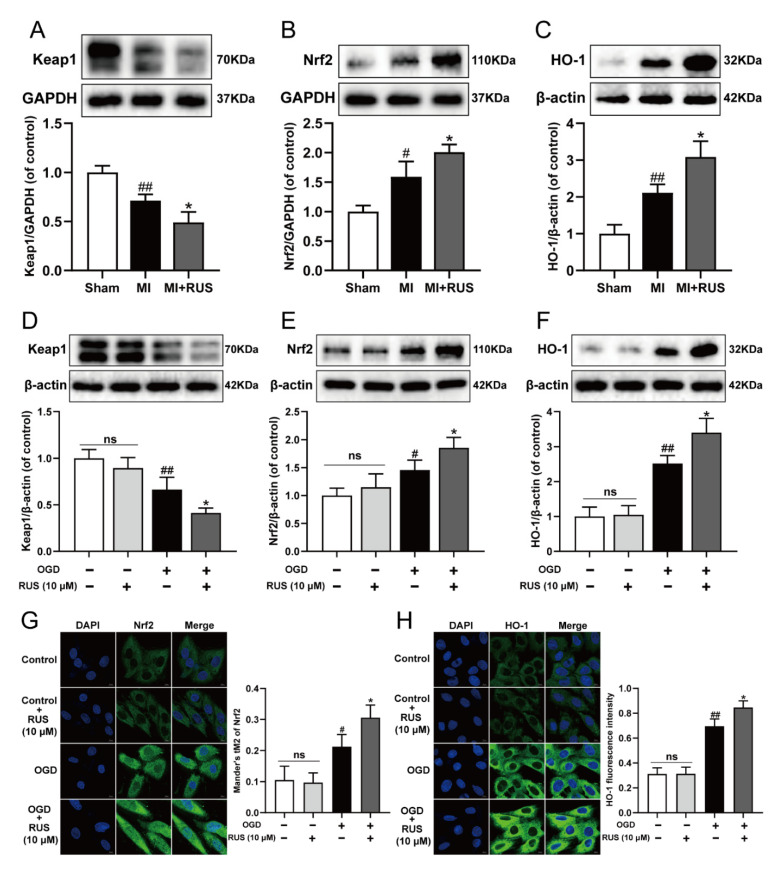
RUS significantly activated the Keap1/Nrf2/HO-1 signaling pathway in MI. Western blotting analysis of (**A**) Keap1, (**B**) Nrf2 and (**C**) HO-1 in the heart tissues of sham, MI and MI treated with 0.75 mg/kg RUS groups (*n* = 3). Western blotting analysis of (**D**) Keap1, (**E**) Nrf2 and (**F**) HO-1 in H9c2 cardiomyocytes (*n* = 3–4). Immunofluorescence images of (**G**) Nrf2 and (**H**) HO-1 in H9c2 cardiomyocytes (scale bar, 10 µm, *n* = 3). Results were expressed as mean ± SD. ^#^ *p*  <  0.05, ^##^ *p*  <  0.01 vs. the sham or control group, * *p*  <  0.05 vs. the MI or OGD group.

Then, we investigated whether RUS could regulate the Keap1/Nrf2/HO-1 signaling pathway in MI. As shown in Figure 7A–C, 0.75 mg/kg RUS treatment dramatically induced the down-regulation of Keap1 and up-regulation of Nrf2 and HO-1 in MI mice. Additionally, in in vitro experiments, RUS treatment down-regulated the expression of Keap1, increased the level of Nrf2 and HO-1, and enhanced the nucleus accumulation of Nrf2 in OGD-injured H9c2 cardiomyocytes (Figure 7D–H). However, RUS did not affect the Keap1/Nrf2/HO-1 pathway in H9c2 cardiomyocytes without OGD injury.

### 3.8. BCAT1 or BCAT2 Knockdown Partially Weakened the Protective Effect of RUS in OGD-Injured H9c2 Cardiomyocytes

**Figure 8:**
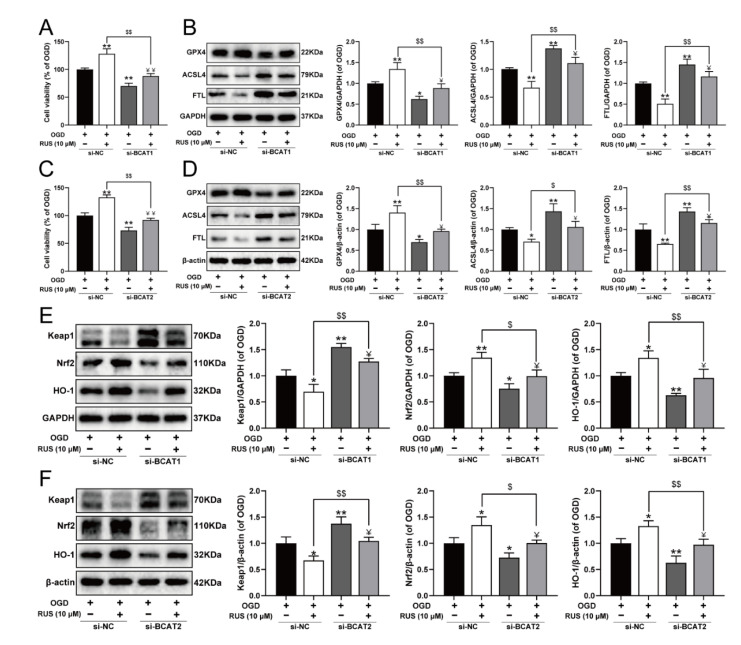
BCAT1 or BCAT2 knockdown partially weakened the protective effect of RUS in OGD-injured H9c2 cardiomyocytes. (**A**) The cell viability of H9c2 cardiomyocytes after being transfected with BCAT1 siRNA (*n* = 6). (**B**) Western blotting analysis of GPX4, ACSL4, and FTL in H9c2 cardiomyocytes transfected with BCAT1 siRNA (*n* = 3). (**C**) The cell viability of H9c2 cardiomyocytes after being transfected with BCAT2 siRNA (*n* = 6). (**D**) Western blotting analysis of GPX4, ACSL4, and FTL in H9c2 cardiomyocytes transfected with BCAT2 siRNA (*n* = 3). Western blotting analysis of Keap1, Nrf2 and HO-1 in H9c2 cardiomyocytes transfected with (**E**) BCAT1 siRNA or (**F**) BCAT2 siRNA (*n* = 3). Results were expressed as mean ± SD. * *p*  <  0.05, ** *p*  <  0.01 vs. the OGD group, ^$^ *p*  <  0.05, ^$$^ *p * <  0.01 vs. the group treated with RUS and OGD, ^Ұ^ *p*  <  0.05, ^ҰҰ^ *p * <  0.01 vs. the group treated with OGD and BCAT1 siRNA or BCAT2 siRNA.

To further investigate whether RUS ameliorated MI in a BCAT1 or BCAT2-dependent manner, the siRNA transfection experiment was carried out. As exhibited in Figure 8, RUS conferred a repressive effect against ferroptosis, and activated the Keap1/Nrf2/HO-1 pathway in OGD-injured H9c2 cardiomyocytes. However, BCAT1 or BCAT2 siRNA significantly reduced the viability of RUS-treated cardiomyocytes with OGD injury, and further aggravated ferroptosis by decreasing GPX4, as well as increasing the expression of ACSL4 and FTL in OGD-injured cardiomyocytes with RUS treatment (Figure 8A–D). Additionally, compared with RUS-treated cardiomyocytes with OGD injury, BCAT1 or BCAT2 siRNA inhibited the Keap1/Nrf2/HO-1 signaling pathway, as evidenced by the significantly decreased expression of Nrf2 and HO-1 and the upregulation of Keap1 (Figure 8E,F). All of these results indicate that BCAT1 and BCAT2 played an important role in mediating the protective actions of RUS.

## 4. Discussion

RUS is a natural product with anti-inflammatory, anti-thrombotic and antioxidant characteristics [24]. Many previous studies focused on its treatment of various inflammatory diseases and cerebral ischemic injury [16,17]. However, there are few reports about the therapeutic effects of RUS on heart diseases. In the present study, the therapeutic actions of RUS on myocardial ischemia were investigated. The results demonstrate that RUS reduced infarct size and cardiac tissue damage in MI mice, and improved cell viability and antioxidant capacity in OGD-injured H9c2 cardiomyocytes. All of these results reveal RUS as a promising option for the treatment of MI.

Many clinical studies suggested that the level of myocardial iron is a prognostic factor of CVDs [25,26], and ferroptosis is an iron-mediated mechanism with pathological effects in the heart [4]. Several studies reported that ferroptosis inhibitors such as ferrostatin-1, compound 968, and iron chelator desferrioxamine are conducive to ameliorating ischemia-reperfusion injury [27,28]. Lipid peroxidation and iron are necessary for the execution of ferroptosis. ACSL4 is involved in the biosynthesis and remodeling of polyunsaturated fatty acid-PEs in cellular membranes, which influences lipid composition [29]. FTL is a ferritin responsible for the storage of iron and greatly impacts iron metabolism in the cell [30]. In addition, GPX4 is known for specifically catalyzing the reduction of lipid peroxides, thereby removing lipid ROS [7]. Thus, several indexes, including iron, GPX4, ACSL4, FTL and lipid peroxides, were used to comprehensively evaluate the inhibitory effect of RUS on myocardial ferroptosis. Functionally, RUS effectively protected against MI-induced ferroptosis.

Subsequently, the metabolic changes between MI and RUS mice were revealed through an HPLC-Q-TOF/MS-based metabolomic analysis method. In the current research, RUS significantly increased the level of L-arginine and taurine. Pharmacological studies have demonstrated an impairment of L-arginine transport in patients with heart failure and restorative actions of supplemental L-arginine on endothelial dysfunction and vascular function in heart failure [31,32]. Taurine is an abundant amino acid with a high concentration in the heart. It was reported to play a central role in ischemia-reperfusion injury [33,34]. Additionally, the levels of allantoin, methionine, methionine sulfoxide, and phenylacetylglycine were lower than those in the MI group. Allantoin is created by non-enzymatic oxidation of uric acid by scavenging ROS, which may provide a biomarker for chronic heart failure [35]. High levels of methionine and methionine sulfoxide can act as metabolic toxins in atherosclerosis [36]. Meanwhile, studies suggested that the heightened phenylacetylglycine might increase incident CVD risks through enhancing platelet responsiveness and thrombosis potential [37]. All of these studies indicated a close association with the development of cardiovascular diseases and suggested the therapeutic effect of RUS for MI. Subsequently, the metabolic pathways and regulatory enzymes were further analyzed. Histidine metabolism, valine, leucine and isoleucine biosynthesis were screened out as the most important metabolic pathways. Histidine metabolism is associated with several physiological functions including cell differentiation, proliferation and hematopoiesis, and it regulates inflammation in the process of CVDs and atherosclerosis [38]. Valine, leucine and isoleucine biosynthesis pathway regulate the level of branched-chain amino acids, which are essential for the generation of macromolecule precursors and energy for ATP production in TCA cycle [39]. Additionally, the regulatory enzymes branched-chain amino acid aminotransferase 1/2 (BCAT1/BCAT2) were highly ranked in the results of KEGG pathway enrichment, suggesting that BCAT1 and BCAT2 might be the main functional proteins that RUS exerted a protective effect on in MI mice. In particular, RUS was verified to promote the expression of BCAT1 and BCAT2 during MI by the experiments.

The canonical role of BCAT1/BCAT2 includes transamination and redox regulation, performing a variety of physiological functions, including cell proliferation, oxidoreductase activity, and autophagy regulation [39,40]. The function of BCAT1 and BCAT2 have been widely reported in various types of cancers, such as gliomas and chronic myeloid leukaemia [41,42]. Moreover, BCAT2 was identified as a specific inhibitor of ferroptosis by the mechanism of regulating intracellular glutamate levels in liver and pancreatic cancer cells [43]. Prior to this study, we elucidated that cardiac overexpression of BCAT1/BCAT2 inhibited MI-induced ferroptosis by means of the down-regulation of ferritin and the up-regulation of GPX4 [23]. Nevertheless, the specific mechanism of BCAT1/BCAT2 in regulating ferroptosis during MI remains undefined. In the present study, the knockdown of BCAT1 or BCAT2 significantly aggravated ferroptosis. Meanwhile, Keap1 was activated, while Nrf2 and HO-1 were restrained in H9c2 cardiomyocytes with BCAT1 or BCAT2 knockdown. Conversely, cardiac overexpression of BCAT1/BCAT2 promoted the Keap1/Nrf2/HO-1 pathway. Keap1/Nrf2/HO-1 is an important signaling pathway implicated in defense mechanisms against oxidative stress [9,10]. This pathway coordinates the antioxidant defensive system in the regulation of ferroptosis, and regulates many genes responsible for modulating ferroptosis, including glutathione regulation, NADPH regeneration, iron regulation, formation of ROS, and mitochondrial function [44,45]. It was reported that the activated p62-Keap1-Nrf2 antioxidant pathway caused by TRIM21 deficiency protected mice from CAL surgery, and alleviated doxorubicin-induced ferroptosis [46]. Similarly, HO-1 inhibitor Znpp reversed the protective effect of icariin on hypoxia/reoxygenation-induced ferroptosis [47]. All of these studies revealed the important role of the Keap1/Nrf2/HO-1 pathway in alleviating ferroptosis during CVDs. Here, we found that levels of BCAT1 and BCAT2 positively regulated the Keap1/Nrf2/HO-1 pathway, which suggested that BCAT1 and BCAT2 effectively inhibited ferroptosis via the activation of the Keap1/Nrf2/HO-1 pathway in MI. Then, we investigated the effect of RUS on the Keap1/Nrf2/HO-1 signaling pathway. Our findings suggest that RUS contributed to the down-regulation of Keap1 and the promotion of Nrf2 and HO-1. Therefore, the protective effect of RUS may be associated with the promotion of the Keap1/Nrf2/HO-1 pathway.

Additionally, to further explore the role of BCAT1/BCAT2 in the cardioprotection of RUS, transfections were performed using BCAT1 siRNA and BCAT2 siRNA. The results demonstrate that knockdown of BCAT1 or BCAT2 impaired the inhibitory effect of RUS on ferroptosis in OGD-injured H9c2 cardiomyocytes. Meanwhile, knockdown of BCAT1 or BCAT2 also degraded the ameliorative effect of RUS on the Keap1/Nrf2/HO-1 signaling pathway. This evidence confirmed that BCAT1 and BCAT2 were essential proteins for RUS to exert an inhibitory effect on myocardial ferroptosis. Additionally, it also revealed that RUS could activate the Keap1/Nrf2/HO-1 pathway through the positive regulation of BCAT1 and BCAT2. However, this study also had some limitations. Although these results demonstrate the effects of RUS on myocardial ferroptosis associated with BCAT1 and BCAT2, further validation is required using BCAT1-knockout or BCAT2-knockout mice, and other regulatory enzymes that might exert crucial roles in the metabolic pathways of RUS remain to be validated in future works. 

## 5. Conclusions

The present study confirmed that RUS exerted cardioprotective effects and inhibited MI-induced ferroptosis. The potential mechanism is associated with enhancing the levels of BCAT1/BCAT2 and the activation of the Keap1/Nrf2/HO-1 signaling pathway. Moreover, we found that BCAT1 and BCAT2 could alleviate ferroptosis via the activation of the Keap1/Nrf2/HO-1 signaling pathway during MI. Our study elucidated an undiscovered mechanism for the treatment of MI, as well as a novel pharmacologic action and molecular mechanisms of RUS.

## Figures and Tables

**Table 1:** KEGG pathway enrichment results of regulatory enzymes.

Enrichment Pathway	Observed Gene Count	Background Gene Count	Matching Proteins in Our Network
Histidine metabolism	5	16	ALDH3A1, ALDH1A3, DDC, ABP1, HRH2
Valine, leucine and isoleucine biosynthesis	3	8	BCAT1, BCAT2, IARS, LARS2
Aminoacyl-tRNA biosynthesis	5	48	CCBL1, GMPS, TGM3, BCAT1, BCAT2
D-Glutamine and D-glutamate metabolism	2	6	CCBL1, GMPS, TGM3
Neomycin, kanamycin and gentamicin biosynthesis	1	2	GCK, HK3, SI
Arginine biosynthesis	2	14	GLS2, GLS
Glycine, serine and threonine metabolism	3	33	MAOB, MAOA, CEL, GAMT, GATM
Pentose and glucuronate interconversions	2	18	TKTL1, RPE, TKTL2
Pyrimidine metabolism	3	39	NT5C1A, NT5C, NT5M
Purine metabolism	4	65	XDH, HPRT1, PNP, APRT, HPRT1, GDA
Pentose phosphate pathway	2	22	PGM1, PGM2, PNP, UPP1
Nitrogen metabolism	1	6	CCBL1, GMPS, TGM3
Galactose metabolism	2	27	GCK, HK3, SI
Taurine and hypotaurine metabolism	1	8	GAD1, CSAD, GLRA1
Cysteine and methionine metabolism	2	33	CTH, MTR, MTHFR, TAT
Caffeine metabolism	1	10	XDH, HPRT1, PNP
Amino sugar and nucleotide sugar metabolism	2	37	KHK, GAA, MGAM, GANC
Arginine and proline metabolism	2	38	GAMT, GATM
Valine, leucine and isoleucine degradation	2	40	BCAT1, BCAT2, IARS
Nicotinate and nicotinamide metabolism	1	15	QPRT, KMO
Starch and sucrose metabolism	1	18	GCK, HK3, SI

## Data Availability

Data are contained within the article.

## Supplementary Materials

The following supporting information can be downloaded at: https://www.mdpi.com/article/10.3390/antiox11030583/s1, Figure S1: PCA score plots obtained from the analysis of the QC group and other groups; Figure S2: Changes of the expression of GPX4 and ACSL4 with the prolongation of OGD-injured time in H9c2 cardiomyocytes; Figure S3: Enrichment analysis of metabolic pathway and regulatory enzymes; Figure S4: Changes of the expression of BCAT1 and BCAT2 with the prolongation of OGD-injured time in H9c2 cardiomyocytes; Figure S5: The relevant statistical results of immunohistochemistry analysis of BCAT1 in Figure 4A and the relevant statistical results of immunohistochemistry analysis of BCAT2 in Figure 4B; Figure S6: The relevant statistical results of immunohistochemistry analysis of Keap1, Nrf2 and HO-1. Table S1: The RSD of retention time and peak area in QC samples; Table S2: Sequences of siRNAs; Table S3: The parameters for assessing the model quality of OPLS-DA; Table S4: Identification of 58 differential metabolites in urine; Table S5: Identification of 4 differential metabolites in serum.
