# Supplementary material for: Ruscogenin Alleviates Myocardial Ischemia-Induced Ferroptosis through the Activation of BCAT1/BCAT2
Source: Antioxidants (Basel). 2022 Mar 18;11(3):583. doi: 10.3390/antiox11030583 (PMC8945524; doi:10.3390/antiox11030583)
Supplement: Supplementary file 1 [file antioxidants-11-00583-s001.zip › antioxidants-1596330-supplementary.pdf]

Supplementary Information

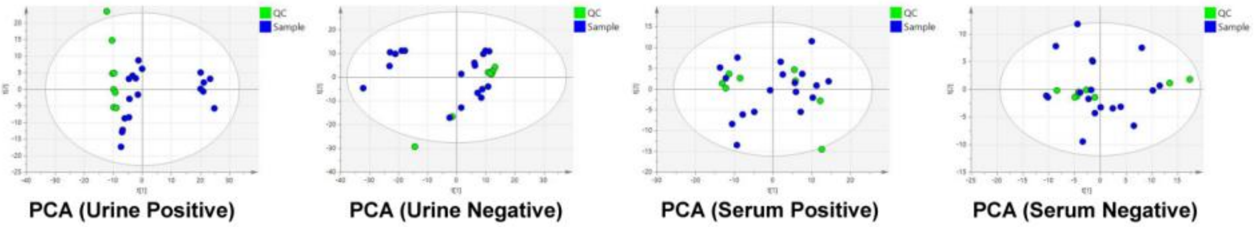

Supplementary Figure S1. PCA score plots obtained from the analysis of the QC group (green dots) and other groups (Sham, MI, and MI treated with 0.75 mg/kg RUS: blue dots), based on Q-TOF system (n=6).

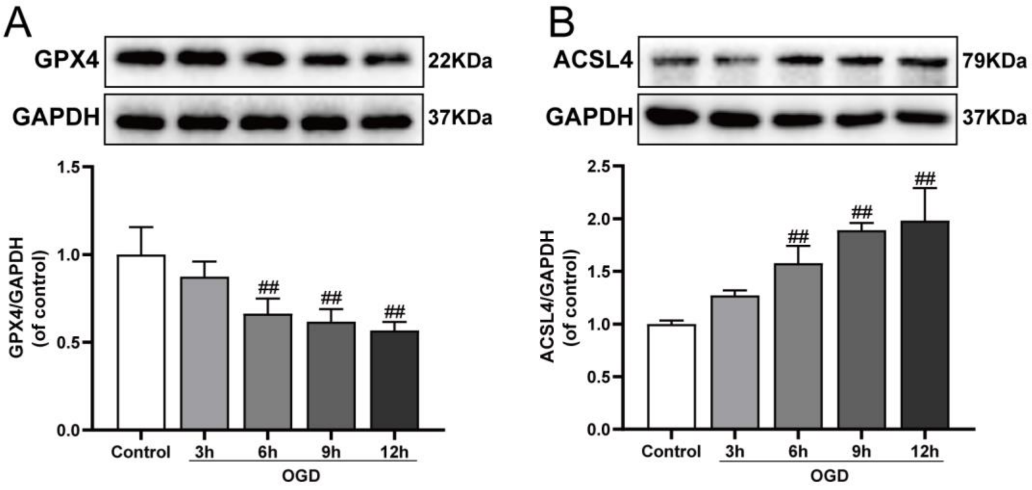

Supplementary Figure S2. Changes of the expression of **(A)** GPX4 and **(B)** ACSL4 with the prolongation of OGD-injured time in H9c2 cardiomyocytes (n=3-4). Results were expressed as mean ± SD. $^{\#\#}p < 0.01$ vs. the control group.

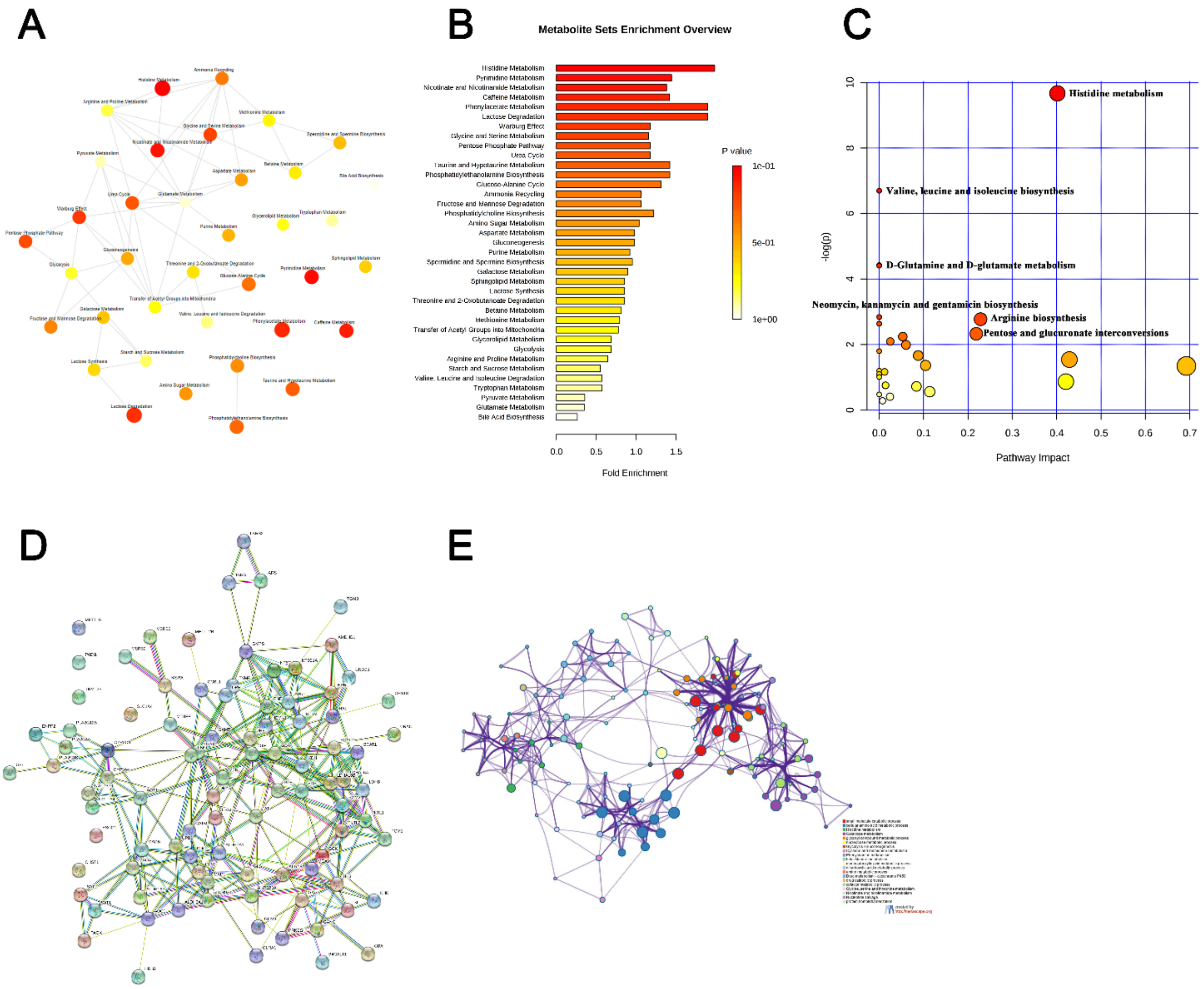

Supplementary Figure S3. Enrichment analysis of metabolic pathway and regulatory enzymes. (A) Network map of metabolic pathway. **(B)** Overview of pathways related to the differential endogenous metabolites. **(C)** A summary of pathway analysis by MetPA. **(D)** Network map of regulatory proteins. **(E)** GO enrichment analysis of regulatory enzymes.

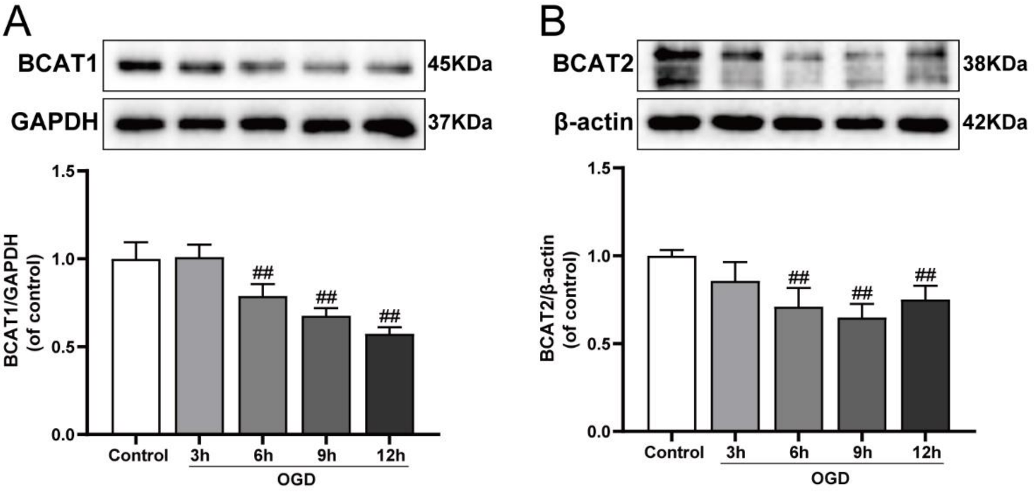

Supplementary Figure S4. Changes of the expression of **(A)** BCAT1 and **(B)** BCAT2 with the prolongation of OGD-injured time in H9c2 cardiomyocytes (n=3-4). Results were expressed as mean ± SD. $^{\#\#}p < 0.01$ vs. the control group.

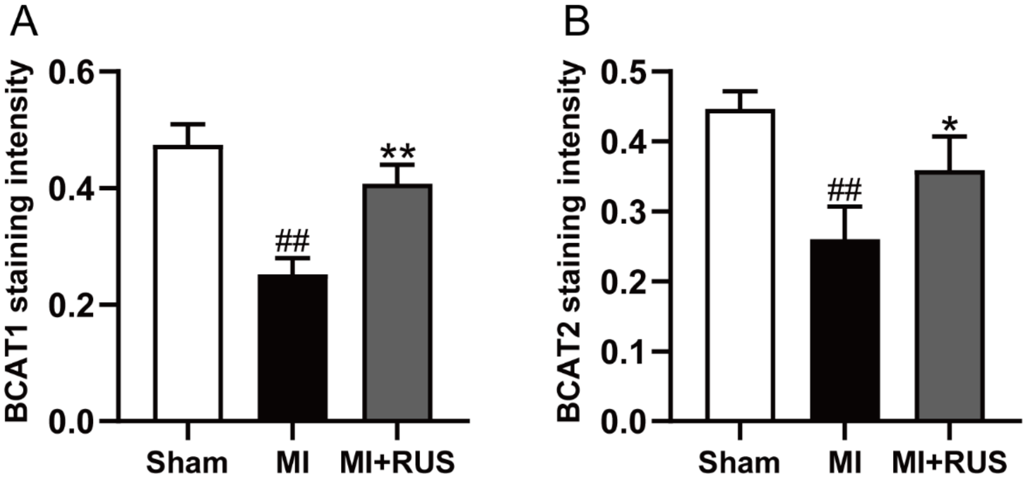

Supplementary Figure S5. (A) The relevant statistical results of immunohistochemistry analysis of BCAT1 in Figure 4A (n=3). **(B)** The relevant statistical results of immunohistochemistry analysis of BCAT2 in Figure 4B (n=3). Results were expressed as mean ± SD. $^{\#\#}p < 0.01$ vs. the sham group, $^{*}p < 0.05$, $^{**}p < 0.01$ vs. the MI group.

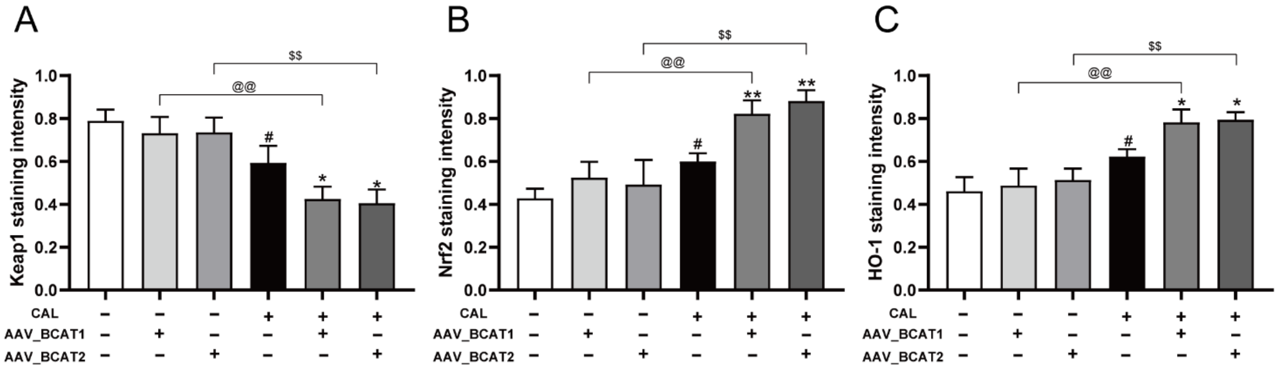

Supplementary Figure S6. The relevant statistical results of immunohistochemistry analysis of **(A)** Keap1, **(B)** Nrf2 and **(C)** HO-1 in Figure 6C (n=3). Results were expressed as mean ± SD. $^{\#}p <$ 0.05 vs. the sham group, $^{*}p < 0.05$, $^{**}p < 0.01$ vs. the MI group, $^{@@}p < 0.01$ vs. the group treated with AAV-BCAT1, $^{\$\$}p < 0.01$ vs. the group treated with AAV-BCAT2.

Supplementary Table S1. The RSD of retention time and peak area in QC samples

Sample-mode	RSD of retention time (%)	RSD of peak area (%)
Urine-positive	0.67	3.71
Urine-negative	0.45	3.94
Serum-positive	0.36	4.21
Serum-negative	0.33	4.74

Supplementary Table S2. Sequences of siRNAs

Gene name	Accession number	siRNA sequence (5' to 3')
BCAT1	NM_017253.3	Forward: GGCUGCAACUAUGGAUCUU<br>Reverse: AAGAUCCAUAGUUGCAGCC
BCAT2	NM_022400.2	Forward: GGAGUGGAACAGCAAGACA<br>Reverse: UGUCUUGCUGUUCCACUCC

Supplementary Table S3. The parameters for assessing the model quality of OPLS-DA

Sample-mode	R^2X (cum)	R^2Y (cum)	Q2
Urine-positive	0.698	0.997	0.984
Urine-negative	0.669	0.955	0.964
Serum-positive	0.786	0.998	0.875
Serum-negative	0.736	0.995	0.905

Supplementary Table S4. Identification of 58 differential metabolites in urine

Ion Mode	Identification	m/z	Rt (min)	VIP	p-value	FC[b]	Metabolic pathways	Enzymes	Genes
	N-butyrylglycine	748.53	2.37	1.07	3.82E-04	1.22		Glycine N-acyltransferase; Glycine N-acyltransferase-like protein 1; Glycine N-acyltransferase-like protein 2	GLYAT; GLYAT1; GLYAT2
	(3-Arylcarbonyl)-alanine	226.14	2.36	1.41	3.93E-07	0.60			
	Methylimidazoleacetic acid	261.18	14.89	1.08	1.09E-03	1.48	Histidine Metabolism	Aldehyde dehydrogenase, dimeric NADP-preferring; Aldehyde dehydrogenase family 1 member A3	ALDH3A1;ALDH1A3
	2-Deoxysepiapterin	204.09	13.90	1.21	8.52E-04	0.70		Glycine N-acyltransferase; Glycine N-acyltransferase-like protein 1; Glycine N-acyltransferase-like protein 2	GLYAT; GLYAT1; GLYAT2
	2-Methylbutyrylglycine	256.15	2.27	1.15	1.16E-02	0.94			
	2-Methylguanosine	112.05	10.69	1.42	4.69E-11	1.75			
Positive	3-Methylguanine	369.15	10.57	1.31	3.06E-06	1.54			
	3'-O-Methylguanosine	277.12	10.93	1.31	2.53E-05	1.34			
	4-Guanidinobutanoic acid	204.13	4.03	1.20	1.95E-06	1.17			
	Allantoin	60.08	5.21	1.03	4.40E-04	1.21		Putative 2-oxo-4-hydroxy-4-carboxy-5-ureidoimidazoline decarboxylase	PRHOXNB
	L-Arginine	189.09	16.35	1.31	2.39E-04	0.88			
	Cellotriose	344.13	16.47	1.20	1.58E-02	0.91		Glucosidase 2 subunit beta;Glucosylceramidase; Cytosolic beta-glucosidase	PRKCSH; GBA
	Creatine	250.16	15.09	1.37	2.74E-06	1.85	Glycine and Serine Metabolism; Arginine and Proline Metabolism	Guanidinoacetate N-methyltransferase; Glycine amidinotransferase, mitochondrial	GAMT; GATM;

Glycylprolylhydroxyproline	323.14	16.82	1.26	3.51E-04	0.88			
Guanine	451.16	14.01	1.42	3.88E-08	0.70	Purine Metabolism	Adenine phosphoribosyltransferase; Hypoxanthine-guanine phosphoribosyltransferase; Guanine deaminase	APRT; HPRT1; GDA
Hexanoylglycine	233.09	2.39	1.12	8.50E-06	1.17		Glycine N-acyltransferase; Glycine N-acyltransferase-like protein 1; Glycine N-acyltransferase-like protein 2	GLYAT; GLYAT1; GLYAT2
Histamine	150.06	14.32	1.43	7.45E-11	0.59	Histidine Metabolism	Aromatic-L-amino-acid decarboxylase; Amiloride-sensitive amine oxidase; Retina-specific copper amine oxidase; Histamine H2 receptor	DDC; ABP1; HRH2
Imidazoleacetic acid	307.12	6.81	1.02	2.08E-02	0.81	Histidine Metabolism	Alpha-aminoadipic semialdehyde dehydrogenase; Aldehyde dehydrogenase family 1 member A3	ALDH9A1; ALDH1A3
Isobutyrylglycine	818.61	2.39	1.38	4.96E-08	1.83		Glycine N-acyltransferase; Glycine N-acyltransferase-like protein 1; Glycine N-acyltransferase-like protein 2	GLYAT; GLYAT1; GLYAT2
L-Alloisoleucine	149.11	4.66	1.35	3.28E-04	0.75			
Ureidoisobutyric acid	173.09	15.85	1.39	7.21E-08	1.42	Pyrimidine Metabolism	Dihydropyrimidinase; Beta-ureidopropionase	DPYS; UPB1
L-Glutamine	147.08	15.96	1.40	6.39E-08	0.77	Pyrimidine Metabolism; Glutamate Metabolism; Purine Metabolism	Kynurenine--oxoglutarate transaminase 1; GMP synthase; Protein-glutamine gamma-glutamyltransferase E	CCBL1; GMPS; TGM3
L-Isoleucine	396.16	4.26	1.31	2.88E-04	0.70	Valine, Leucine and Isoleucine Degradation	Branched-chain-amino-acid aminotransferase, cytosolic; Branched-chain-amino-acid aminotransferase, mitochondrial; Isoleucine--tRNA ligase, cytoplasmic	BCAT1; BCAT2; IARS;
L-Leucine	226.08	14.02	1.23	7.33E-03	0.87	Valine, Leucine and Isoleucine Degradation	Branched-chain-amino-acid aminotransferase, cytosolic; Branched-chain-amino-acid aminotransferase, mitochondrial; Probable leucine--tRNA ligase, mitochondrial	BCAT1; BCAT2; LARS2

	L-Norleucine	76.08	4.58	1.22	6.97E-05	1.18			
	Methionine	162.11	6.23	1.34	4.17E-07	3.57	Betaine Metabolism; Methionine Metabolism	Methionine synthase; Methylenetetrahydrofolate reductase; Tyrosine aminotransferase	MTR; MTHFR; TAT
	N1-Acetylspermidine	398.03	14.51	1.07	3.99E-03	1.79		Diamine acetyltransferase 2; Diamine acetyltransferase 1; Peroxisomal N(1)-acetyl-spermine/spermidine oxidase	SAT2; SAT1; PAOX
	N-Acetyllactosamine	365.11	15.72	1.04	7.11E-05	1.16		Carbohydrate sulfotransferase 3; Carbohydrate sulfotransferase 1; Beta-1,4-galactosyltransferase 1	CHST3; CHST1; B4GALT1;
	N-Methyl-4-pyridone-3-carboxamide	240.16	3.28	1.12	6.94E-05	1.24	Nicotinate and Nicotinamide Metabolism	Aldehyde oxidase	AOX1
	Phenylacetylglycine	820.62	2.43	1.33	2.35E-06	1.59		Glycine N-acyltransferase; Glycine N-acyltransferase-like protein 1; N-acetyltransferase ESCO1	GLYAT; GLYAT1; ESCO1
	Ribothymidine	399.20	15.31	1.27	5.96E-05	1.73		tRNA (uracil(54)-C(5))-methyltransferase homolog	TRMT2B
	Thymine	127.05	6.72	1.30	2.28E-03	2.16	Pyrimidine Metabolism	Dihydropyrimidine dehydrogenase; Thymidine phosphorylase	DPYD; TYMP
	Tryptophyl-Alanine	223.09	10.73	1.44	5.45E-13	2.57			
	Tyramine glucuronide	336.11	16.56	1.43	1.57E-12	1.89		UDP-glucuronosyltransferase 2B28; UDP-glucuronosyltransferase 2B4; UDP-glucuronosyltransferase 1-4	UGT2B28; UGT2B24; UGT1A4
	Urocanic acid	155.13	5.21	1.13	9.73E-05	1.26	Histidine Metabolism; Ammonia Recycling	Histidine ammonia-lyase; Urocanate hydratase	HAL; UROC1
	Xanthurenic acid	414.30	3.07	1.36	3.49E-07	1.70	Tryptophan Metabolism	HemK methyltransferase family member 1; Methyltransferase-like protein 2B; Methyltransferase-like protein 6	HEMK1; METTL2B; METTL6
Negative	2-Hydroxyethanesulfonate	325.11	12.40	1.43	8.51E-05	0.52			

4-Imidazolone-5-propionic acid	74.02	15.36	1.33	2.48E-03	0.83	Histidine Metabolism	Urocanate hydratase; Probable imidazolonepropionase	UROC1; AMDHD1
5-Acetylamino-6-amino-3-methyluracil	151.06	13.66	1.03	1.07E-04	1.21	Caffeine Metabolism		
Aminoacetone	251.05	15.62	1.40	3.91E-07	1.76	Glycine and Serine Metabolism	Amine oxidase [flavin-containing] B; Amine oxidase [flavin-containing] A; Bile salt-activated lipase	MAOB; MAOA; CEL
D-Glutamine	487.19	16.00	1.39	2.82E-05	0.68		Glutaminase liver isoform, mitochondrial; Glutaminase kidney isoform, mitochondrial	GLS2; GLS
Fructose	397.07	13.25	1.03	3.81E-03	1.55	Amino Sugar Metabolism; Fructose and Mannose Degradation; Galactose Metabolism	Ketohexokinase; Lysosomal alpha-glucosidase; Maltase-glucoamylase, intestinal; Neutral alpha-glucosidase C	KHK; GAA; MGAM; GANC
Homocysteinesulfinic acid	324.96	3.07	1.53	5.29E-15	13.07			
Aspartate-4-phosphate	363.02	1.79	1.38	6.19E-04	0.88			
L-Galactose	357.03	14.08	1.44	1.32E-07	2.02			
Glucose	179.06	14.20	1.24	1.61E-03	0.69	Glycolysis; Galactose Metabolism; Gluconeogenesis	Glucokinase; Hexokinase-3; Sucrase-isomaltase, intestinal	GCK; HK3; SI
L-Threonine	118.05	15.63	1.26	1.10E-02	0.84	Glycine and Serine Metabolism	Threonine--tRNA ligase, cytoplasmic; L-serine dehydratase/L-threonine deaminase; Neutral amino acid transporter A	TARS; SDS; SLC1A4
Methionine sulfoxide	302.10	16.05	1.22	1.60E-05	1.35	Methionine Metabolism	Mitochondrial peptide methionine sulfoxide reductase; Methionine-R-sulfoxide reductase B2, mitochondrial; Methionine-R-sulfoxide reductase B3	MSRA; MSRB2; MSRB3
Paraxanthine	183.00	12.91	1.23	5.50E-03	0.83	Caffeine Metabolism	Xanthine dehydrogenase/oxidase; Cytochrome P450 3A4; Cytochrome P450 2C9	XDH; CYP3A4; CYP2C9

p-Cresol sulfate	349.00	1.69	1.41	4.71E-08	1.64			
Quinolinic acid	277.04	3.11	1.28	1.41E-02	0.90	Nicotinate and Nicotinamide Metabolism	Nicotinate-nucleotide pyrophosphorylase; Kynurenine 3-monooxygenase	QPRT; KMO
Ribose 1-phosphate	165.04	3.06	1.49	2.70E-05	0.41	Pentose Phosphate Pathway; Purine Metabolism	Phosphoglucomutase-1; Phosphoglucomutase-2; Purine nucleoside phosphorylase; Uridine phosphorylase 1	PGM1; PGM2; PNP; UPP1
Taurine	288.98	14.69	1.43	9.56E-06	0.80	Taurine and Hypotaurine Metabolism; Bile Acid Biosynthesis	Glutamate decarboxylase 1; Cysteine sulfinic acid decarboxylase; Glycine receptor subunit alpha-1	GAD1; CSAD; GLRA1
Thiocysteine	126.91	2.44	1.34	9.94E-06	1.83		Cystathionine gamma-lyase	CTH
Threonic acid	116.03	6.13	1.42	6.34E-06	0.80			
Uridine	367.07	13.67	0.00	1.46E+00	4.26	Pyrimidine Metabolism	Cytosolic 5'-nucleotidase 1A; 5'(3')-deoxyribonucleotidase, cytosolic type; 5'(3')-deoxyribonucleotidase, mitochondrial	NT5C1A; NT5C; NT5M
Xanthine	151.02	4.64	1.51	3.62E-08	1.66	Purine Metabolism	Xanthine dehydrogenase/oxidase; Hypoxanthine-guanine phosphoribosyltransferase; Purine nucleoside phosphorylase	XDH; HPRT1; PNP
Xylulose 5-phosphate	245.02	3.07	1.43	2.05E-05	0.71	Pentose Phosphate Pathway	Transketolase-like protein 1; Ribulose-phosphate 3-epimerase; Transketolase-like prote 2	TKTL1; RPE; TKTL2

RT(min)[a]: retention time; **FC[b]**: fold change, as calculated by average relative quantitation acquired from MI group/RUS group, and a value more than 1 indicates an increased level in the metabolites of MI group.

Supplementary Table S5. Identification of 4 differential metabolites in serum

Ion Mode	Identification	m/z	Rt (min)	VIP	p-value	FC[b]	Metabolic pathways	Enzymes	Genes
Positive	LysoPE(0:0/16:1(9Z))	279.23	13.25	2.14	1.48E-03	0.78		Ectonucleotide pyrophosphatase/phosphodiesterase family member 2	ENPP2
Negative	Glyceraldehyde	711.34	6.9869	1.99	2.23E-07	1.62	Glycerolipid Metabolism; Fructose and Mannose Degradation; Glycerolipid Metabolism	Aldose reductase; 4-trimethylaminobutyraldehyde dehydrogenase; Alpha-aminoadipic semialdehyde dehydrogenase	AKR1B1; ALDH9A1; ALDH7A1
	L-Lactic acid	160.90	2.14	1.45	4.20E-03	1.91	Gluconeogenesis; Pyruvate Metabolism	L-lactate dehydrogenase A-like 6A; L-lactate dehydrogenase B chain; M-L-lactate dehydrogenase A-like 6B	LDHAL6A; LDHB; LDHAL6B
	PE(22：2(13Z，16Z)/ 15：0)	343.24	13.67	1.26	1.54E-03	0.86		Cytosolic phospholipase A2; Phospholipase A2; Group XIIB secretory phospholipase A2-like protein	PLA2G4A; PLA2G1B; PLA2G12B

RT(min)[a]: retention time; **FC[b]**: fold change, as calculated by average relative quantitation acquired from MI group/RUS group, and a value more than 1 indicates an increased level in the metabolites of MI group.
